# Protocol for a preclinical systematic review and meta-analysis of pharmacological targeting of peroxisome proliferator-activated receptors in experimental renal injury

**DOI:** 10.1136/bmjos-2021-100240

**Published:** 2021-11-15

**Authors:** William P Martin, Yeong H D Chuah, Emer Conroy, Alison L Reynolds, Conor Judge, Francisco J López-Hernández, Carel W le Roux, Neil G Docherty

**Affiliations:** 1 Diabetes Complications Research Centre, School of Medicine, Conway Institute of Biomolecular and Biomedical Research, University College Dublin, Belfield, Dublin 4, Ireland; 2 Biomedical Facility, Agriculture and Food Science Building, University College Dublin, Belfield, Dublin 4, Ireland; 3 School of Veterinary Medicine, University College Dublin, Belfield, Dublin 4, Ireland; 4 Conway Institute of Biomolecular and Biomedical Research, University College Dublin, Belfield, Dublin 4, Ireland; 5 HRB Clinical Research Facility, National University of Ireland Galway, Galway, Ireland; 6 Translational Medical Device Lab, National University of Ireland Galway, Galway, Ireland; 7 Instituto de Investigación Biomédica de Salamanca (IBSAL) and Instituto de Estudios de Ciencias de la Salud de Castilla y León (IECSCYL), Paseo de San Vicente, 58-182 - Hospital Virgen de la Vega, Planta 10^a^, 37007, Salamanca, Castilla y León, Spain; 8 Diabetes Research Group, Ulster University, Coleraine, UK

## Abstract

**Introduction:**

Impaired lipid metabolism in the renal tubule plays a prominent role in the progression of renal fibrosis following acute kidney injury (AKI) and in chronic kidney disease (CKD). Peroxisome proliferator-activated receptors (PPARs) are promising druggable targets to mitigate renal fibrosis by redirecting metabolism, including restoration of fatty acid oxidation (FAO) capacity. We aim to synthesise evidence from preclinical studies of pharmacological PPAR targeting in experimental renal injury, and inform the design of future studies evaluating PPAR-mediated restoration of FAO in AKI and CKD.

**Methods and analysis:**

Studies reporting on the impact of pharmacological PPAR modulation in animal models of renal injury will be collected from MEDLINE (Ovid), Embase and Web of Science databases. Predefined eligibility criteria will exclude studies testing medications which are not specific ligands of one or more PPARs and studies involving multimodal pharmacological treatment. The Systematic Review Centre for Laboratory Animal Experimentation risk of bias tool and Collaborative Approach to Meta-Analysis and Review of Animal Experimental Studies checklist will be used to assess quality of the included studies. Data extraction will be followed by a narrative synthesis of the data and meta-analysis where feasible. Analysis will be performed separately for AKI, CKD and renal transplant models. Subgroup analyses will be performed based on study design characteristics, PPAR isotype(s) targeted, and classes of PPAR-targeting medications used. Risk of publication bias will be assessed using funnel plotting, Egger’s regression and trim-and-fill analysis.

**Ethics and dissemination:**

Ethical approval is not required. Findings will be published in a peer-reviewed journal and presented at scientific meetings.

**PROSPERO registration number:**

CRD42021265550.

## Introduction

### Background

#### Acute kidney injury and chronic kidney disease: inter-relationships and associated morbidity

Chronic kidney disease (CKD) is a growing public health problem, affecting 9.1% of the global population in 2017,^1^ intertwined with the rising prevalence of obesity, type 2 diabetes mellitus (T2DM) and hypertension.^2^ While morbidity and mortality rates for other non-communicable diseases have declined over the past three decades, no such favourable trends have been reported for CKD.^3^ Despite recent advances in the treatment of CKD, most notably sodium-glucose cotransporter-2 (SGLT2) inhibitors,^4,5^ it remains a progressive disease for most patients and additional strategies are required to abrogate the residual risk of renal functional decline.^4^ Furthermore, acute kidney injury (AKI) rates are high in people with and without pre-existing CKD, and the presence of T2DM, the leading cause of CKD, further amplifies these associations.^6^ Multiple AKI episodes are associated with cumulative risk for a range of adverse consequences, including accelerated renal functional decline, advanced CKD and increased risk of cardiovascular disease.^7,8^

#### Impairment of fatty acid oxidation in AKI and CKD

Fatty acid oxidation (FAO) is impaired in AKI and CKD and contributes to tubular dedifferentiation and the development of tubulointerstitial fibrosis.^9–11^ This mechanism of progressive renal functional decline is not directly addressed by existing therapies which modify the course of CKD, including SGLT2 inhibitors and renin–angiotensin–aldosterone system blockade.^12–14^ SGLT2 inhibitors do however promote a shift towards increased peripheral fatty acid utilisation which may synergise with directed pharmacological stimulation of FAO to further reduce progression of renal fibrosis.^15^ No antifibrotic therapies are currently used in the treatment of AKI sequelae or CKD in clinical practice.^16^ However, given the well-characterised deficit in tubular FAO in CKD and its mechanistic association with the development and progression of renal fibrosis,^9^ peroxisome proliferator-activated receptor (PPAR)-directed therapies may be considered as strong candidates in this regard.^16^

#### PPARs and their pharmacological activation

PPARs are transcription factors belonging to the superfamily of nuclear receptors that regulate genes involved in lipid and glucose metabolism.^17^ Three isotypes of PPARs exist (alpha, beta/delta and gamma) which differ in their tissue distribution and ligand specificity.^18^ PPARα is highly expressed in metabolically active tissues including liver and kidney, PPARγ is preferentially expressed in adipose tissues, while PPARβ/δ is expressed ubiquitously across most tissue types.^19^ Both PPARα and PPARβ/δ activate FAO.^20–22^ PPARγ regulates fatty acid storage in adipose tissues and adipocyte differentiation, and contributes to insulin sensitivity.^20,23^ Drug classes such as fibrates (PPARα agonists indicated for dyslipidaemia) and thiazolidinediones (TZDs; PPARγ agonists indicated for T2DM) target PPARs.^24^ Moreover, selective PPAR modulators as well as dual and pan-PPAR agonists have been developed for application in metabolic diseases such as dyslipidaemia and T2DM, as well as downstream end-organ complications including cardiovascular disease and non-alcoholic fatty liver disease.^25–28^

#### Clinical consequences of pharmacological PPAR modulation in kidney diseases

Compared with other end-organ complications of metabolic diseases, fewer clinical studies have evaluated the potential value of PPAR modulation in kidney diseases, perhaps due to concerns regarding reversible increases in serum creatinine following fibrate initiation as well as oedema and heart failure risks following TZD treatment.^29,30^ For example, no randomised controlled studies of fibrates with a primary renal outcome have been performed in people with T2DM. Nevertheless, the potential benefits of PPARα agonism in diabetic kidney disease (DKD) were highlighted by a post-hoc analysis of the Action to Control Cardiovascular Risk in Diabetes (ACCORD) Lipid Trial (NCT00000620).^31^ Randomisation to fenofibrate (n=2636) was associated with slower decline in estimated glomerular filtration rate (eGFR) and lower incident albuminuria compared with placebo (n=2632).^31^ Similar findings were reported in the Fenofibrate Intervention and Event Lowering in Diabetes (FIELD) study (ISRCTN64783481), in which randomisation to fenofibrate (n=4895) was associated with preservation of 5 mL/min/body surface area eGFR over 5-year follow-up compared with randomisation to placebo (n=4900) in patients with T2DM.^32^

#### PPARs as potential therapeutic targets for renal fibrosis

Studies linking loss of tubular FAO with renal fibrosis have to date been principally focused on mitochondrial metabolism,^33^ although peroxisomes are likely of strategic importance to maintenance of tubular FAO given their particularly high density in proximal tubular cells^34,35^ as well as the mandatory requirement for peroxisomal β-oxidation to chain-shorten fatty acids prior to mitochondrial transfer for oxidative phosphorylation.^36^ Furthermore, PPARα is highly expressed in proximal tubular cells^19^ and reduced proximal tubular PPARα expression underpins the FAO deficit which promotes renal fibrosis.^37–39^ Restoration of tubular FAO through PPARα or PPARα/β dual agonism reduces renal injury in experimental models of renal fibrosis.^40,41^

Although PPARγ is considered to play a less prominent role in the direct stimulation of FAO compared with the other PPAR isotypes,^17^ it does nevertheless play a crucial role in the maintenance of renal metabolic homoeostasis and is involved in renal lipid and glucose metabolism as well as systemic blood pressure regulation.^42^ For example, Lyu *et al* demonstrated that PPARγ controls tubular substrate utilisation for energy generation by reducing epidermal growth factor-stimulated glycolysis in the proximal tubule, thereby favouring metabolism of lipids as the predominant energy source in this tubular segment.^43^ Multiple preclinical studies of PPARγ agonism via TZD treatment have demonstrated antifibrotic effects in experimental models of diabetic and non-diabetic renal fibrosis.^44–48^ Such findings lend credence to the concept that PPARγ agonism may be renoprotective in patients with and without diabetes.^49^ However, preclinical studies have not been uniformly positive and equipoise remains^50^.

Pharmacological PPARα and/or PPARβ/δ modulation thus offers an attractive means of promoting FAO to oppose renal fibrosis,^39,41^ with potential for translatability to human AKI and CKD. PPARγ agonism also has potential to favourably redirect renal metabolism to mitigate renal fibrosis.^42^ Renoprotective effects derived from pharmacological activation of two or more PPAR isotypes has been reported and warrants further evaluation.^51,52^ Furthermore, repurposing of licenced drugs that activate PPARs, including fibrates, TZDs and glitazars (dual PPARα/γ agonists), for a renal fibrosis indication could decrease time and cost associated with the drug development pipeline.^16,24^ Indeed, as TZD treatment reduced renal cyst growth in rodent models of polycystic kidney disease, a recent phase 1b cross-over study tested the safety of low-dose pioglitazone in patients with autosomal dominant polycystic kidney disease.^53^ Treatment with pioglitazone 15 mg for 1 year did not significantly reduce total kidney volume but was found to be as safe as placebo.^53^ Larger randomised studies are planned to more definitively evaluate its effects on total kidney volume.^53^ No human clinical trials examining pharmacological stimulation of FAO to mitigate renal fibrosis have yet been conducted.^16^

However, a crucial intermediary step prior to the conduct of a human clinical trial of pharmacological PPAR modulation to modify CKD progression is a systematic and rigorous evaluation of the efficacy of this approach in preclinical models, including assessment of external and construct validity.^54^ This systematic review and meta-analysis will synthesise evidence from the multiple preclinical studies performed to test the impact of pharmacological PPAR targeting in experimental renal injury, and inform the design of future preclinical and clinical studies evaluating FAO restoration through PPAR modulation in the treatment of AKI sequelae and CKD.

### Objectives

This systematic review will aim to:

1. Summarise the impact of pharmacological PPAR targeting in experimental acute and chronic renal injury.
2. Investigate if effects on renal injury vary based on PPAR isotype targeted (PPARα vs PPARβ/δ vs PPARγ vs dual PPAR activation vs pan-PPAR activation).
3. Summarise the mechanistic mediators of renoprotection investigated by authors following pharmacological PPAR targeting.
4. Summarise reporting of safety outcome measures following pharmacological PPAR activation (hepatotoxicity, cardiotoxicity, carcinogenicity, mortality).

## Methods and Analysis

### Registration and reporting

This protocol was formulated using the Systematic Review Centre for Laboratory Animal Experimentation (SYRCLE) template format.^55^ A preprint version of this protocol was registered with Open Science Framework, available at: https://osf.io/vscm4k/.^56^ The present protocol was also preregistered with PROSPERO.^57^ We will adhere to the recommended checklist for reporting preclinical systematic reviews and meta-analyses when publishing our findings to improve the reporting quality, transparency and reproducibility of our systematic review.^58^

### Research question

The research question was formulated according to SYRCLE guidance^59^ in a style similar to the PICO format as follows :

► Intervention: Treatment with an agent that pharmacologically activates one or more PPAR as its primary mechanism of action.
► Disease of interest: Kidney disease (AKI, CKD or renal transplantation).
► Population: Animal models.
► Outcome measures: Renal injury and function measures (detailed below and in table 1).

The research question is thus phrased as: ‘What is the effect of pharmacological targeting of PPARs on renal injury in animal models of kidney disease?’

### Outcome measures

#### Primary outcome measures

Primary outcome measures pertain to renal injury and function indices and include:

1. Plasma/serum creatinine.
2. Plasma/serum urea or blood urea nitrogen (BUN).
3. Creatinine clearance.
4. Glomerular filtration rate.
5. Urinary albumin/protein excretion; in studies that report multiple measurements of urinary protein excretion, only one will be included in the final analysis, with the default being urinary albumin-to-creatinine ratio where available.
6. Histological parameters of renal injury, including standardised reporting of glomerular histological changes and injury scores.
7. Kidney size, including kidney weight or kidney weight/ body weight ratio.
8. Kidney cyst size, including kidney cyst volume or kidney cystic index.

#### Secondary outcome measures

Secondary outcome measures are subcategorised into four groups as follows:

1. Mechanisms underpinning renal responses to pharmacological PPAR targeting investigated by authors, including renal fibrosis, renal inflammation, renal oxidative stress, renal apoptosis, renal epithelial-to-mesenchymal transition, renal lipotoxicity and glucotoxicity and renal nicotinamide adenine dinucleotide metabolism.
2. Urinary biochemical evidence of renal injury (biomarkers), including, for example, neutrophil gelatinase-associated lipocalin and kidney injury molecule-1.
3. Metabolic outcome measures, including body weight, glycaemia, dyslipidaemia and blood pressure.
4. Safety outcome measures, including hepatotoxicity, cardiotoxicity, carcinogenicity and mortality.

### Search strategy

#### Electronic search strategy

Ovid MEDLINE, Embase via Embase.com and Web of Science Core Collection will be used for the systematic literature search. Peer-reviewed journal articles published from database inception to July 2021 will be retrieved. A detailed search strategy has been compiled in accordance with best practice,^59^ outlined in tables 2–4. The search strategy was developed in collaboration with a librarian at University College Dublin, Ireland. Search components (SCs) were built around the first three components of the research question outlined above (intervention, disease of interest and study population) to retrieve relevant studies through systematic searching.^59^ The three categories of SCs are outlined according to this order in tables 2–4. For MEDLINE and Embase searches, the three SCs combine standardised subject terms (Medical Subject Headings for MEDLINE, Emtree for Embase) with free-text searches in the title, abstract and author-supplied keywords using Boolean Logic (‘OR’). Records retrieved by the Embase.com search will be limited to those in the Embase database which are not present in MEDLINE. For the Web of Science search, only free-text searching in the title, abstract and author-supplied keywords will be performed as no thesaurus terms exist in this database.

##### SC1: pharmacological PPAR targeting

Studies examining pharmacological PPAR targeting will be retrieved through custom SCs designed to retrieve studies mentioning PPARs in the context of agonism, modulation, stimulation or activation by free-text searching. Elements of this SC were adapted from Liu and Wang.^60^ Free-text searches for all PPARs, the three PPAR isotypes (α, β/δ, and γ) and dual/pan-PPAR activity will be related to truncated versions of agonist, modulator, stimulator, stimulant and activator by proximity searching such that both terms (eg, ‘PPAR’ and ‘agonist’) are mandated to occur within five words of each other. Furthermore, free-text search terms for drug classes including fibrates, TZDs, selective PPAR modulators and glitazars, as well as individual constituent medications contained therein, will be included along with searching for individual PPARα and pan-PPAR agonists. Pharmacological activation of fatty acid metabolism or oxidation will also be included in the free-text search. Additionally, standardised subject terms for PPARs, fibrates, and TZDs will be included in MEDLINE and Embase.

##### SC2: kidney disease

Studies examining kidney injury will be retrieved using search terms adapted from prior preclinical systematic reviews of interventions for experimental renal injury.^61,62^ The following modifications were made to the search strategy devised by Mihajlovic *et al* ^61^:

1. Addition of standardised subject terms specific to DKD in MEDLINE and Embase.
2. Addition of free-text search terms for ‘acute kidney injury’, ‘acute renal injury’, ‘chronic kidney disease’, ‘chronic renal disease’, ‘diabetic kidney disease’, ‘diabetic renal disease’, ‘diabetic nephropathy’, ‘nephropathy’ and ‘kidney insufficiency’.
3. Incorporation of the UK English spelling of ‘ischaemia’ for the terms ‘kidney ischemia’ and ‘renal ischemia’.

The plural terms for ‘kidney/renal injury’ and ‘nephropathy’ were not included as these terms are typically used in the singular to refer to the clinical syndromes of either AKI or CKD. Where multiple renal insults are modelled in the preclinical setting, these generally converge on either an AKI or CKD model, which are comprehensively captured by other elements of the SC for kidney diseases. Abbreviations for ‘AKI’ and ‘CKD’ were not included as free-text terms due to concerns about their non-specificity, while the kidney disease SCs outlined in tables 2–4 comprehensively cover the retrieval of acute and chronic renal injury studies in the databases queried. There is some redundancy in the kidney disease free-text terms employed; for example, studies mentioning ‘acute kidney injury’ in the title, abstract or keywords would already be captured by ‘kidney injury’. However, the free-text terms outlined are retained to improve transparency and comprehension of the kidney disease SC.

##### SC3: animal models

Studies in animal models will be retrieved by adapting MEDLINE via PubMed and Embase via Ovid animal search filters generated by the SYRCLE.^63–65^ The MEDLINE via PubMed search filter was adapted for MEDLINE via Ovid, while the Embase via Ovid search filter was adapted for Embase via Embase.com. The free-text element of the MEDLINE via PubMed search filter was also adapted to retrieve studies involving animal models in Web of Science.

##### Overall search strategy: combining the three individual SCs

The three SCs will subsequently be combined with the Boolean logic term ‘AND’ to retrieve potentially relevant studies for prescreening of titles and abstracts. The overall search can thus be summarised as: SC1 (pharmacological PPAR targeting) AND SC2 (kidney disease) AND SC3 (animal models). No restrictions will be placed on language or publication date.

The search strategy was piloted in July 2021 and resulted in the retrieval of over 2500 studies, decreasing to just over 1750 studies following removal of duplicate entries. Preliminary progress with title and abstract screening indicates that approximately 25% of screened studies will be included for full-text review, suggesting that the literature search successfully identified relevant studies.

#### Other sources for study identification

Reference lists of included original studies as well as relevant review articles retrieved by the systematic search will be manually reviewed to search for additional preclinical studies of pharmacological PPAR activation with a kidney outcome measure. If the study is not a duplicate of a study retrieved by the electronic literature search and it is deemed potentially suitable following review of the title and abstract, the full-text of the study will be reviewed.

### Study selection

After searching the literature, records will be exported to the reference manager EndNote V.X9 (Clarivate) and merged. Duplicate records will be removed using the R package ASySD,^66^ generated by the Collaborative Approach to Meta-Analysis and Review of Animal Experimental Studies (CAMARADES) group, and manually verified for accuracy.

Thereafter, prescreening of titles and abstracts against predefined eligibility criteria will be performed for all records. If the abstract is not available or not informative enough, the article full-text will be screened.

Full-text screening will be performed for studies deemed potentially eligible after review of the title and abstract. If full-text articles are not available or accessible, corresponding authors will be contacted via email with a request to supply the full text. The reason(s) for article exclusion will be recorded and a Preferred Reporting Items for Systematic Reviews and Meta-Analyses flow diagram documenting the process of study selection completed. Article screening will be performed via Systematic Review Facility (SyRF; CAMARADES).^67^ Default SyRF parameters will be used for article screening such that each study will be independently screened by two reviewers, with disagreements resolved by a third reviewer. WPM, YHDC, EC, AR, FJL-H and NGD will perform title and abstract screening. WPM, YHDC, EC, AR, CJ, FJL-H and NGD will perform full-text screening.

Study eligibility criteria are listed below in the order in which they will be applied during article screening. Study eligibility criteria are also stratified by whether they will be applied at the title and abstract plus full-text screening stages or just the full-text screening stage alone. All criteria applied during title and abstract screening will also be applied during full-text screening. A list of eligibility criteria broken down in PICO format is available in the PROSPERO record, which is also available on Open Science Framework at: https://osf.io/vscm4k/.^56^

#### Inclusion criteria

##### Title and abstract plus full-text screening stages

1. Original research studies in animal models of kidney disease (AKI, CKD or renal transplantation). Studies will not be excluded on the basis of modality of kidney disease induction, provided that one or more of the outcomes relating to kidney injury or function listed below are reported.
2. Treatment with an agent which pharmacologically targets one or more PPAR as its primary mechanism of action, including the PPARα, PPARβ/δ or PPARγ receptors as well as agents targeting two or all three PPAR isotypes (ie, with dual and pan-PPAR activity). One or more outcomes relating to kidney injury or function reported, including:
  – Plasma/serum creatinine.
  – Plasma/serum urea or BUN.
  – Creatinine clearance.
  – Glomerular filtration rate.
  – Urinary albumin/protein excretion.
  – Kidney histological parameters.
  – Kidney size, including kidney weight or kidney weight/body weight ratio.
  – Kidney cyst size, including kidney cyst volume or kidney cystic index.

##### Full-text screening stage only

► Presence of an age-matched and time-matched control group treated with placebo/vehicle.

#### Exclusion criteria

##### Title and abstract plus full-text screening stages

1. Non-original research articles, including reviews, systematic reviews, meta-analyses, conference proceedings, editorials, commentaries and patent applications.
2. Exclusively human studies.
3. Exclusively in vitro, ex vivo or in silico studies.
4. No outcomes relating to kidney injury or function available. Studies which test the impact of pharmacological PPAR modulation on other aspects of renal disease, such as nephrolithiasis, renal cell cancer or coagulopathy associated with nephrotic syndrome, but which do not report any of the kidney injury or function measures detailed above will be excluded.
5. Absence of treatment with an agent which pharmacologically targets one or more PPAR isotype as its primary mechanism of action.

##### Full-text screening stage only

1. Treatment with a drug which increases PPAR activity but which is not a specific ligand of one or more PPARs (ie, interventions that indirectly implicate PPARs as potential therapeutic agents for kidney disease will be excluded; only studies which examine agents with direct pharmacological activity at one or more PPARs as their primary mechanism of action will be included).
2. Multimodal pharmacological treatment with two or more agents, including therein an agent to increase PPAR activity, whereby unique effects of the PPAR-targeting pharmacological agent cannot be directly determined.
3. Less than three animals in treatment/intervention group(s).
4. Absence of an age-matched and time-matched control group treated with placebo/vehicle.
5. Irretrievable manuscript full-texts.

### Assessment of study quality and risk of bias

Study quality and risk of bias will be assessed in studies deemed eligible following full-text review. Study quality will be assessed using an adapted version of the CAMARADES study quality checklist,^68^ including appraisal of the following components: publication in a peer-reviewed journal, reporting the species/strain of animals in the title or abstract and in the full-text, provision of author conflict of interest statements, statement of compliance with animal welfare regulations, reporting basic animal and housing characteristics, reporting of sample size calculation, statement on randomisation of treatment allocation, reporting concealment of treatment allocation, statement on blinded outcome assessment and reporting criteria for inclusion and/or exclusion of data. Articles will receive a point for compliance with each item in the checklist and summary study quality scores will be reported for each item. Risk of bias will be assessed using the SYRCLE risk of bias tool, consisting of 10 items evaluating for selection, performance, detection, attrition, reporting and other biases.^69^ Each item in the risk of bias tool will be recorded as ‘yes’ (low risk of bias), ‘no’ (high risk of bias) or ‘unclear’ (insufficient details present to appropriately assess the risk of bias).^69^ Risk of bias and study quality assessment will be independently performed by two reviewers, with disagreements resolved by a third reviewer. WPM, YHDC, EC, AR, CJ, FJL-H and NGD will perform study quality and risk of bias assessments.

### Data extraction

#### Methods for data extraction

Data extraction will be performed using the SyRF platform (https://syrf.org.uk/) for studies deemed eligible following full-text review.^67^ Data extraction will be performed using the following steps:

1. Direct extraction of data from results text, tables and figures.
2. Extraction of data from graphs using WebPlotDigitizer software or the metaDigitise R package.^70,71^
3. Contact the corresponding author by email for original data if it is not reported or cannot be extracted from the article full-text. In the event of missing data whereby there are no corresponding author contact details, or no response from authors within 3 weeks from initial email despite a reminder email being sent in the interim, the study will be omitted from meta-analysis. If an article is excluded from meta-analysis due to missing data, other aspects of the primary article may still be included in the systematic review, such as study design characteristics, study quality and risk of bias, which will be extracted and summarised in a narrative synthesis.

All continuous data will be recorded as mean±SD or median (IQR). SEM will be recalculated to SD as follows: SD=√n×SEM. In the event that the number of animals is unclear, a conservative estimate will be made. Where data are reported as median (IQR), the corresponding author will be contacted for raw data.

One reviewer (WPM) will extract the data. A second reviewer (NGD) will check the data extracted by WPM for inconsistencies, including assessment of whether or not the data for individual variables falls within expected ranges as well as inspection for outlying data points which will be manually verified. Furthermore, independent data extraction will be performed for a randomly sampled subset of 10% of the included full-texts by YHDC, EC, AR, CJ, FJL-H and NGD to assess the accuracy of data extraction by WPM. This independently extracted data will not be used for meta-analysis but will be compared against that extracted by WPM. Agreement between reviewers will be calculated using Cohen’s kappa index, κ, for qualitative data and the intraclass correlation coefficient for quantitative data. Inter-rater reliability for the randomly sampled 10% of full-texts will be reported in the systematic review. If the level of inter-rater reliability is acceptable, the data extracted by WPM will be used for meta-analysis; if inter-rater reliability is poor, fully independent data extraction will be performed by two reviewers, with discrepancies resolved by a third reviewer.

For AKI models, data from the time point demonstrating greatest efficacy after administration of the PPAR-targeting agent will be used for primary analyses. Where an outcome is measured at multiple time points after intervention, measurements at early (<24 hours), intermediate (24-72 hours) and late time points (≥72 hours) will be recorded. For CKD and renal transplant models, data from the latest time point after administration of the PPAR-targeting agent will be used for primary analyses. Where an outcome is measured at multiple time points after intervention, measurements at early (<4 weeks), intermediate (4-7 weeks) and late time points (≥8 weeks) will be recorded.

#### Data to be extracted

The data to be extracted for both study characteristics and for primary and secondary outcome measures is summarised in table 1.

### Data analysis

A descriptive summary, including visualisations, of animal models employed, PPAR isotype(s) targeted and PPAR-targeting drug classes used, as well as changes in renal injury parameters across all included studies will be prepared. Furthermore, this descriptive summary will also be stratified by the three major kidney disease models studied: AKI, CKD and renal transplant models. Data from acute-on-chronic renal injury models will be incorporated with data from AKI models. For each stream of analysis (AKI, CKD and renal transplant), depending on the comparability of outcome measures as well as the quality and amount of available evidence identified, a meta-analysis will be performed for all kidney outcome measures (outlined in table 1) where feasible.

#### Meta-analysis

Meta-analysis will be conducted for one between-group comparison, namely differences in parameters between the kidney disease control group and the group treated with PPAR-activating pharmacological monotherapy. In the event that a study has multiple intervention groups, some or all of which include groups treated with PPAR-activating medications, only data from intervention group(s) treated with PPAR-activating drug monotherapy will be extracted and meta-analysed. Continuous outcomes that are measured consistently across studies and expressed in natural units will be estimated as weighted mean difference.^72^ For other continuous outcome measures with more heterogeneous measurement methods and/or units, results will be grouped and reported as the standardised mean difference.^73^ Random-effects models will be used for meta-analysis in the likely event that moderate or high levels of heterogeneity are observed between the included studies, thereby accounting for both within-study and between-study variance.^74^ The I^2^ index will be the metric used to assess heterogeneity between studies.^75^ Meta-analysis will be performed using the R programming language in RStudio.^76^

#### Meta-analytical power and required number of studies

**Figure 1:**
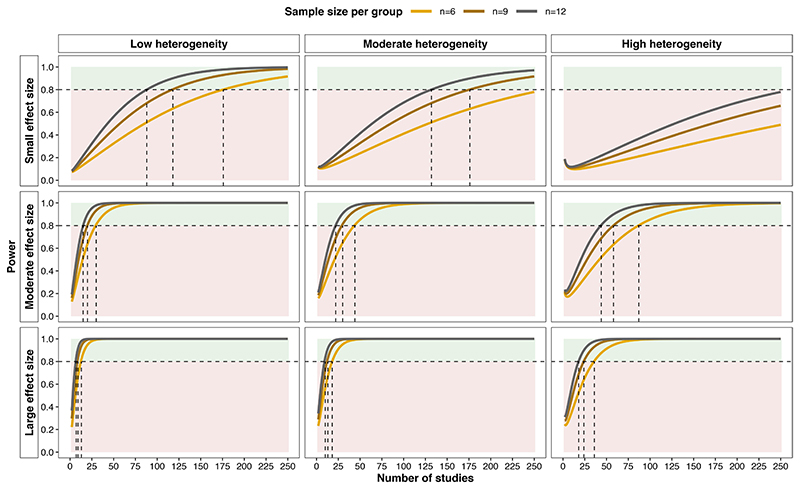
Power calculation for overall effect of pharmacological PPAR activation on experimental renal injury by random-effects meta-analysis. The plot illustrates the number of studies required for adequate power with a two-tailed alpha of 0.05 stratified by anticipated effect size, level of heterogeneity between studies and sample size per group. The meta-analytical power calculation was performed using the function ‘mpower’ from the R package metapower.^77^ Heterogeneity was defined on the basis of the I^2^ index as follows: 0.25 (low), 0.5 (moderate) and 0.75 (high). Effect size was defined on the basis of Cohen’s d as follows: 0.2 (small), 0.5 (moderate) and 0.8 (large). The horizontal dashed lines indicate statistical power (1-β) of 0.8. Areas of green shading correspond to power ≥0.8, while red shading corresponds to power <0.8. Vertical dashed lines illustrate the number of studies required to achieve statistical power of 0.8 for each of the three sample sizes per group. Statistical power is presented across the range of 2–250 studies as based on piloting of the search strategy, the number of included full-texts is expected to fall within this range. PPAR, peroxisome proliferator-activated receptor.

A power calculation was performed using the function ‘mpower’ from the R package metapower^77^ to calculate the number of studies required for random-effects meta-analysis with a two-tailed alpha of 0.05, stratified by effect size, the degree of heterogeneity between studies and sample size per group (figure 1). Adequate statistical power (≥0.8) will be difficult to achieve if the overall effect of pharmacological PPAR activation on renal injury is small and the degree of heterogeneity between studies is moderate or large. However, even with moderate or large heterogeneity between studies and an average sample size per group of n=6, if the effect of pharmacological PPAR activation on renal injury is moderate or large then the meta-analysis should be adequately powered with less than 100 studies.

Meta-analysis will be performed separately for models of AKI and CKD as these are distinct clinical syndromes; thus, the number of studies required for adequate meta-analytical power outlined in figure 1 must be considered separately for AKI and CKD models as these will constitute parallel rather than integrated streams of analysis. Accordingly, meta-analysis will be performed for each specific kidney outcome measure which is reported on in ≥20–25 studies of either AKI or CKD models. This number of studies appears feasible following the literature search and initial progress with title and abstract screening, and may also render adequate meta-analytical power despite moderate or high levels of heterogeneity between studies (figure 1). While studies in renal transplantation models may also be considered as a separate meta-analytical stream, it is unlikely that a sufficient number of such studies will be retrieved and their findings are more likely to be summarised in a descriptive fashion.

#### Subgroup analyses

The following characteristics will be examined as a potential source of heterogeneity:

► Animal model characteristics
  – Animal species.
  – Modality of kidney disease induction.
  – Gender (stratified as male vs female vs mixed vs not reported).
  – Comorbidities (obesity vs no obesity, hypertension vs no hypertension, dyslipidaemia vs no dyslipidaemia, diabetes vs no diabetes).
► Pharmacological PPAR targeting
  – PPAR-targeting medication classes (stratified as fibrate vs glitazone vs glitazar vs selective PPAR modulator vs pan-PPAR agonist).
  – PPAR isotype(s) targeted by the drug intervention (stratified as PPARα vs PPARβ/δ vs PPARγ vs two PPARs vs three PPARs).
  – Route of drug administration (stratified as oral vs intravenous vs intraperitoneal vs subcutaneous).
  – Timing of drug treatment (stratified as preventative if given before or during kidney disease induction, or rescue if given after kidney disease induction).
► Reporting of measures to reduce the risk of bias (stratified by results from the SYRCLE risk of bias tool and/ or CAMARADES study quality checklist).

#### Correction for multiple testing

A Bonferroni correction for multiple testing will be applied when interpreting p values from subgroup analyses.^74^ For eight subgroup analyses in studies of AKI, CKD and renal transplantation, this results in a Bonferroni-adjusted p value of 0.006. If one or more subgroup analyses are not performed due to insufficient data, the p value will be adjusted accordingly.

#### Sensitivity analyses

We will assess how timing of primary renal outcome assessment influences effect sizes observed, rather than choosing time points of greatest efficacy (in AKI models) and latest time points assessed (in CKD and renal transplant models). For AKI models, the efficacy of pharmacological PPAR targeting on renal injury measurements obtained at early (<24 hours), intermediate (24–72 hours) and late time points (≥72 hours) will be explored. For CKD and renal transplant models, the efficacy of pharmacological PPAR targeting on renal injury measurements obtained at early (<4 weeks), intermediate (4–7 weeks) and late time points (≥8 weeks) will be explored.

#### Correction for multiple use of a control group

If a study reports data from several experimental groups pertaining to pharmacological PPAR targeting, correction for multiple use of a control group will be performed by dividing the number of animals in the control group by the number of comparisons performed with this control group for groups treated with drugs targeting PPARs.

#### Assessment of publication bias

Funnel plots will be prepared and visually inspected, Duval and Tweedie’s trim-and-fill analysis will be performed and Egger’s test will be performed for kidney outcome measures assessed in ≥20–25 studies,^54,74,78,79^ separately for studies involving AKI, CKD and renal transplant models.

## Discussion

The difficulties in translating preclinical findings to human diseases are well-recognised, and render the drug discovery and development process highly inefficient.^80^ The nephrology community has also struggled to harness promising preclinical findings to improve patient outcomes, perhaps most notably for bardoxolone methyl whose phase 3 evaluation in patients with DKD was terminated early due to high cardiovascular event rates.^81^ Common themes in preclinical research contribute to this translational gap, including issues with reproducibility, limited statistical power and a bias towards publication of positive findings.^80^ Preclinical systematic reviews and meta-analyses offer an opportunity to critically evaluate the strength of interventions in an unbiased fashion and have potential to increase the proportion human clinical trials testing efficacious interventions while simultaneously promoting the 3Rs (replacement, reduction and refinement) in preclinical research.^54^

Limitations of this review include challenges common to all preclinical systematic reviews, namely high heterogeneity between published studies and the existence of publication bias favouring the reporting of positive outcomes which may result in overestimation of treatment effects in meta-analysis.^54^ We will assess included studies for publication bias; furthermore, the absence of language restrictions in our literature search should help to address the existence of reporting bias.^54^ An additional potential limitation of this review is the quality and reporting of the included primary studies. Study quality will be assessed using an adapted version of the CAMARADES study quality checklist,^68^ while risk of bias will be assessed using the SYRCLE risk of bias tool.^69^ Furthermore, planned meta-analytical subgroup analyses will assess study quality and risk of bias measures as potential sources of heterogeneity in the observed effects of pharmacological PPAR targeting on experimental renal injury. Our literature search does not involve a dedicated search of grey literature, and abstracts from recent studies published in conference proceedings will be excluded. Nevertheless, preliminary searches conducted with our existing search strategy indicate the presence of an adequate number of studies for meta-analysis. Furthermore, reference lists of studies retrieved by the systematic literature search will be manually reviewed to search for additional potentially eligible studies, which will increase coverage of the relevant literature.

In summary, this review will systematically evaluate the role of pharmacological PPAR modulation in experimental renal injury. In so doing, we plan to inform the design of future preclinical and clinical studies evaluating FAO restoration through PPAR modulation in renal fibrosis, with potential applications to both human AKI and CKD.

## Figures and Tables

**Table 1:** Data to be extracted from included studies, stratified by study characteristics and outcome measures

Study characteristics	
Category	Description
Study ID	► Authors ► Year ► Journal ► Language
Study design	► Experimental groups, including type of control group(s) ► Number of animals per group ► Duration of follow-up ► Number of experiments and replications
Animal model	► Species ► Strain ► Age ► Gender ► Weight ► Comorbidities: – Obesity—yes/no – Diabetes—yes/no (if yes, type 1 or type 2 diabetes mellitus model) – Dyslipidaemia—yes/no – Hypertension—yes/no ► Kidney disease characteristics: – AKI model vs CKD model vs acute-on-chronic renal injury model vs renal transplant model – Kidney disease manifests as part of natural history or induced – Modality of kidney disease induction
Intervention	► PPAR isotypes pharmacologically targeted: – PPARα – PPARβ/δ – PPARγ – Two PPAR isotypes – Three PPAR isotypes ► Route of administration: – Oral – Intravenous – Subcutaneous – Intraperitoneal ► Dose of PPAR-targeting pharmacological agent ► Timing of intervention – Preventative if given before or during kidney disease induction – Rescue if given after kidney disease induction ► Frequency of intervention (n times per day, once daily, once every n number of days, once weekly) ► Duration of intervention (in days/weeks/months)
Other	► Study quality indicators (adapted from CAMARADES study quality checklist)^68,69^: – Publication in a peer-reviewed journal—yes/no – Species/strain of animals reported in title or abstract and in full-text—yes/no – Provision of author conflict of interest statements—yes/no – Statement of compliance with animal welfare regulations—yes/no – Basic animal and housing characteristics reported—yes/no – Sample size calculation reported—yes/no – Randomisation of treatment allocation reported—yes/no – Concealment of treatment allocation reported—yes/no – Statement on blinded outcome assessment provided—yes/no – Criteria for inclusion and/or exclusion of data provided—yes/no ► Risk of bias assessment using the SYRCLE risk of bias tool – 10 items will be recorded as ‘yes’, ‘no’ or ‘unclear’
**Outcome measures**	
**Category**	**Description (data type; unit)**
Primary	**Renal injury and function indices:** ► Plasma/serum creatinine (continuous; mg/dL or μmol/L) ► Plasma/serum urea or blood urea nitrogen (continuous; mg/dL or μmol/L) ► Creatinine clearance (continuous; mL/min) ► Glomerular filtration rate (continuous; mL/min) ► Urinary albumin/protein excretion (continuous; μg/hour (excretion rate) or μg/mg (normalised to urinary creatinine)) ► Histological parameters of renal injury (continuous; arbitrary units) ► Kidney size parameters (continuous; grams (kidney weight) or percentage (kidney weight/body weight ratio)) ► Kidney cyst size parameters (continuous; mL (kidney cyst volume) or percentage (kidney cystic index)
Secondary	**Mechanistic mediators explored (all categorical; yes/no**) ► Renal fibrosis ► Renal inflammation ► Renal oxidative stress ► Renal apoptosis ► Renal epithelial-to-mesenchymal transition ► Renal lipotoxicity ► Renal glucotoxicity ► Renal nicotinamide adenine dinucleotide metabolism
	**Urinary biochemical evidence of renal injury (biomarkers**) ► Neutrophil gelatinase-associated lipocalin (continuous; μg/hour (excretion rate) or μg/mg (normalised to urinary creatinine)) ► Kidney injury molecule-1 (continuous; μg/hour (excretion rate) or μg/mg (normalised to urinary creatinine))
	**Metabolic parameters** ► Body weight (continuous; g) ► Glycaemia – Circulating glucose (continuous; mg/dL or mmol/L) – HbA_1c_ (continuous; % or mmol/mol) – Fructosamine (continuous; mg/dL or mmol/L) ► Dyslipidaemia (all continuous; mg/dL or mmol/L) – Circulating total cholesterol – LDL-cholesterol – HDL-cholesterol – Triglycerides ► Blood pressure (all continuous; mm Hg) – Systolic blood pressure – Diastolic blood pressure – Mean arterial blood pressure
	**Safety outcomes** ► Hepatotoxicity – Liver enzyme elevation (continuous; mg/dL or mmol/L) – Histological evidence of liver injury (categorical; yes/no) ► Cardiotoxicity – Ejection fraction (continuous; %) – Heart failure (categorical; yes/no) – Histological evidence of cardiac injury (categorical; yes/no) ► Carcinogenicity – Tumour development (categorical; yes/no) – Tumour site (categorical; organ) ► Mortality – Mortality (categorical; yes/no) – Number who died (continuous; n) – Timing of death from start of intervention (continuous; days/weeks/months)

AKI, acute kidney injury; CAMARADES, Collaborative Approach to Meta-Analysis and Review of Animal Data from Experimental Studies; CKD, chronic kidney disease; HbA_1c_, glycated haemoglobin; HDL, high-density lipoprotein; LDL, low-density lipoprotein; PPAR, peroxisome proliferator-activated receptor; SYRCLE, Systematic Review Centre for Laboratory Animal Experimentation.

**Table 2:** Ovid MEDLINE search strategy: Ovid MEDLINE(R) and Epub Ahead of Print, In-Process, In-Data-Review and Other Non-lndexed Citations, Daily and Versions(R)

Search component (number)	Search term type	Search alias	Search content
Pharmacological PPAR Targeting (SC1) Adapted with modifications from Liu and Wang^60^	Free-text	MED_PPAR_FREE	((‘peroxisome proliferator-activated receptor’ OR ‘ppar’) adj5 (agonist* OR modulator* OR stimulat* OR stimulant* OR activat*)).ti,ab,kw. OR
			((‘peroxisome proliferator-activated receptor alpha’ OR ‘ppar alpha’ OR ‘ppar-alpha’ OR ‘pparalpha’ OR ‘ppara’ OR ‘nr1c1’) adj5 (agonist* OR modulator* OR stimulat* OR stimulant* OR activat*)).ti,ab,kw. OR
			((‘peroxisome proliferator-activated receptor beta ‘OR “ppar beta’ OR ‘ppar-beta’ OR ‘pparbeta’ OR ‘pparb’ OR ‘peroxisome proliferator-activated receptor delta’ OR ‘ppar delta’ OR ‘ppar-delta’ OR ‘ppardelta’ OR ‘ppard’ OR ‘nr1c2’) adj5 (agonist* OR modulator* OR stimulat* OR stimulant* OR activat*)).ti,ab,kw. OR
			((‘peroxisome proliferator-activated receptor gamma’ OR ‘ppar gamma’ OR ‘ppar-gamma’ OR ‘ppargamma’ OR ‘pparg’ OR ‘nr1c3’) adj5 (agonist* OR modulator* OR stimulat* OR stimulant* OR activat*)).ti,ab,kw. OR ((‘dual-PPAR’ OR ‘dual PPAR’ OR ‘PPAR-dual’ OR ‘PPAR dual’ OR ‘dual-peroxisome proliferator-activated receptor’ OR ‘dual peroxisome proliferator-activated receptor’ OR ‘peroxisome proliferator-activated receptor-dual’ OR ‘peroxisome proliferator-activated receptor dual’) adj5 (agonist* OR modulator* OR stimulat* OR stimulant* OR activat*)).ti,ab,kw. OR
			((‘pan-PPAR’ OR ‘pan PPAR’ OR ‘PPAR-pan’ OR ‘PPAR pan’ OR ‘pan-peroxisome proliferator-activated receptor’ OR ‘pan peroxisome proliferator-activated receptor’ OR ‘peroxisome proliferator-activated receptor-pan’ OR ‘peroxisome proliferator-activated receptor pan’) adj5 (agonist* OR modulator* OR stimulat* OR stimulant* OR activat*)).ti,ab,kw. OR
			(‘fibrate’ OR ‘fibrates’ OR ‘fibric acid’ OR ‘fibric acids’ OR ‘fibric acid derivative’ OR ‘fibric acid derivatives’ OR ‘bezafibrate’ OR ‘bezafibric acid’ OR ‘ciprofibrate’ OR ‘ciprofibric acid’ OR ‘clinofibrate’ OR ‘aluminium clofibrate’ OR ‘aluminium clofibric acid’ OR ‘clofibrate’ OR ‘clofibric acid’ OR ‘clofibride’ OR ‘choline fenofibrate’ OR ‘choline fenofibric acid’ OR ‘fenofibrate’ OR ‘fenofibric acid’ OR ‘gemfibrozil’ OR ‘simfibrate’).ti,ab,kw. OR
			(‘WY-14,643’ OR ‘WY-14643’ OR ‘WY 14,643’ OR ‘WY 14643’ OR ‘WY14,643’OR ‘WY14643’ OR ‘pirinixic acid’). ti,ab,kw. OR
			(‘selective peroxisome proliferator-activated receptor modulat*’ OR ‘selective PPAR modulât*’ OR ‘SPPAR’ OR ‘SPPARM’ OR ‘SPPARM alpha’ OR ‘SPPARM-alpha’ OR ‘SPPARMalpha’ OR ‘SPPARMa’ OR ‘pemafibrate’ OR ‘K-877’ OR ‘K877’).ti,ab,kw. OR
			(‘thiazolidinedione’ OR ‘thiazolidinediones’ OR ‘glitazone’ OR ‘glitazones’ OR ‘pioglitazone’ OR ‘rosiglitazone’ OR ‘troglitazone’ OR ‘netoglitazone’ OR ‘rivoglitazone’ OR ‘ciglitazone’ OR ‘balaglitazone’ OR ‘darglitazone’ OR ‘edaglitazone’ OR ‘englitazone’ OR ‘lobeglitazone’).ti,ab,kw. OR
			(‘glitazar’ OR ‘glitazars’ OR ‘ragaglitazar’ OR ‘tesaglitazar’ OR ‘muraglitazar’ OR ‘naveglitazar’ OR ‘aleglitazar’ OR ‘saroglitazar’).ti,ab,kw. OR
			(‘lanifibranor’ OR ‘elafibranor’ OR ‘tetradecylthioacetic acid’).ti,ab,kw. OR ((‘fatty acid metabolism’ OR ‘fatty acid oxidation’) adj5 (pharmacol*)).ti,ab,kw.
	Standardised	MED_PPAR_INDEXED	(exp Peroxisome Proliferator-Activated Receptors/ OR exp Fibric Acids/ OR exp Thiazolidinediones/)
	Free-text or standardised	MED_PPAR_COMBINED	MED_PPAR_FREE OR MED_PPAR_INDEXED
Kidney Disease (SC2) Adapted with modifications from Mihajlovic *et al* ^61^ and from Hickson *et al* ^62^	Free-text	MED_KIDNEY_FREE	(‘kidney disease’ OR ‘kidney injury’ OR ‘kidney ischemia’ OR ‘kidney ischaemia’ OR ‘kidney diseases’ OR ‘kidney failure’ OR ‘kidney insufficiency’ OR ‘kidney fibrosis’ OR ‘renal disease’ OR ‘renal diseases’ OR ‘renal injury” OR ‘renal ischemia’ OR ‘renal ischaemia’ OR ‘renal failure’ OR ‘enal insufficiency’ OR ‘renal fibrosis’ OR ‘acute kidney injury’ OR ‘acute renal injury’ OR ‘chronic kidney disease’ OR ‘chronic renal disease’ OR ‘diabetic kidney disease’ OR ‘diabetic renal disease’ OR ‘diabetic nephropathy’ OR ‘nephropathy’).ti,ab,kw.
	Standardised	MED_KIDNEY_INDEXED	(exp kidney diseases/ OR exp acute kidney injury/ OR exp Kidney Failure, Chronic/ OR exp Renal Insufficiency/ OR exp Diabetic Nephropathies/)
	Free-text or standardised	MED_KIDNEY_COMBINED	MED_KIDNEY_FREE OR MED_KIDNEY_INDEXED
Animal Models (SC3)	Free-text	MED_ANIMAL_FREE	SYRCLE MEDLINE via PubMed animal search filter^63^ adapted for MEDLINE via Ovid
	Standardised	MED_ANIMAL_INDEXED	SYRCLE MEDLINE via PubMed animal search filter^63^ adapted for MEDLINE via Ovid
	Free-text or standardised	MED_ANIMAL_COMBINED	MED_ANIMAL_FREE OR MED_ANIMAL_INDEXED
Final Search (SC1 AND SC2 AND SC3)	PPAR, kidney disease and animal model search terms	MED_FINAL	MED_PPAR_COMBINED AND MED_KIDNEY_COMBINED AND MED_ANIMAL_COMBINED

PPAR, peroxisome proliferator-activated receptor; SC, search component; SYRCLE, Systematic Review Centre for Laboratory Animal Experimentation.

**Table 3:** Embase via Embase.com search strategy

Search component (number)	Search term type	Search alias	Search content
Pharmacological PPAR Targeting (SC1) Adapted with modifications from Liu and Wang^60^	Free-text	EMB_PPAR_FREE	((‘peroxisome proliferator-activated receptor’ OR ‘ppar’) NEAR/5 (agonist* OR modulator* OR stimulat* OR stimulant* OR activat*)):ti,ab,kw OR
			((‘peroxisome proliferator-activated receptor alpha’ OR ‘ppar alpha’ OR ‘ppar-alpha’ OR ‘pparaIpha’ OR ‘ppara’ OR ‘nr1c1’) NEAR/5 (agonist* OR modulator* OR stimulat* OR stimulant* OR activât*)):ti,ab,kw OR
			((‘peroxisome proliferator-activated receptor beta’ OR ‘ppar beta’ OR ‘ppar-beta’ OR ‘pparbeta’ OR ‘pparb’ OR ‘peroxisome proliferator-activated receptor delta’ OR ‘ppar delta’ OR ‘ppar-delta’ OR ‘ppardelta’ OR ‘ppard’ OR ‘nr1c2’) NEAR/5 (agonist* OR modulator* OR stimulat* OR stimulant* OR activat*)):ti,ab,kw OR
			((‘peroxisome proliferator-activated receptor gamma’ OR ‘ppar gamma’ OR ‘ppar-gamma’ OR ‘ppargamma’ OR ‘pparg’ OR ‘nr1c3’) NEAR/5 (agonist* OR modulator* OR stimulat* OR stimulant* OR activat*)):ti,ab,kw OR
			((‘dual-PPAR’ OR ‘dual PPAR’ OR ‘PPAR-dual’ OR ‘PPAR dual’ OR ‘dual-peroxisome proliferator-activated receptor’ OR ‘dual peroxisome proliferator-activated receptor’ OR ‘peroxisome proliferator-activated receptor-dual’ OR ‘peroxisome proliferator-activated receptor dual’) NEAR/5 (agonist* OR modulator* OR stimulat* OR stimulant* OR activat*)):ti,ab,kw OR
			((‘pan-PPAR’ OR ‘pan PPAR’ OR ‘PPAR-pan’ OR ‘PPAR pan’ OR ‘pan-peroxisome proliferator-activated receptor’ OR ‘pan peroxisome proliferator-activated receptor’ OR ‘peroxisome pro 1 iterator-activated receptor-pan’ OR ‘peroxisome proliferator-activated receptor pan’) NEAR/5 (agonist* OR modulator* OR stimulat* OR stimulant* OR activat*)):ti,ab,kw OR
			(‘fibrate’ OR ‘fibrates’ OR ‘fibric acid’ OR ‘fibric acids’ OR ‘fibric acid derivative’ OR ‘fibric acid derivatives’ OR ‘bezafibrate’ OR ‘bezafibric acid’ OR ‘ciprofibrate’ OR ‘ciprofibric acid’ OR ‘clinofibrate’ OR ‘aluminium clofibrate’ OR ‘aluminium clofibric acid’ OR ‘clofibrate’ OR ‘clofibric acid’ OR ‘clofibride’ OR ‘choline fenofibrate’ OR ‘choline fenofibric acid’ OR ‘fenofibrate’ OR ‘fenofibric acid’ OR ‘gemfibrozil’ OR ‘simfibrate’):ti,ab,kw OR
			(‘WY-14,643’ OR ‘WY-14643’ OR ‘WY 14,643’ OR ‘WY 14643’ OR ‘WY14,643’ OR ‘WY14643’ OR ‘pirinixic acid’):ti,ab,kw OR
			(‘selective peroxisome proliferator-activated receptor modulat*’ OR ‘selective PPAR modulât*’ OR ‘SPPAR’ OR ‘SPPARM’ OR ‘SPPARM alpha’ OR ‘SPPARM-alpha’ OR ‘SPPARMalpha’ OR ‘SPPARMa’ OR ‘pemafibrate’ OR ‘K-877’ OR ‘K877’):ti,ab,kw OR
			(‘thiazolidinedione’ OR ‘thiazolidinediones’ OR ‘glitazone’ OR ‘glitazones’ OR ‘pioglitazone’ OR ‘rosiglitazone’ OR ‘troglitazone’ OR ‘netoglitazone’ OR ‘rivoglitazone’ OR ‘ciglitazone’ OR ‘balaglitazone’ OR ‘darglitazone’ OR ‘edaglitazone’ OR ‘englitazone’ OR ‘lobeglitazone’):ti,ab,kw OR
			(‘glitazar’ OR ‘glitazars’ OR ‘ragaglitazar’ OR ‘tesaglitazar’ OR ‘muraglitazar’ OR ‘naveglitazar’ OR ‘aleglitazar’ OR ‘saroglitazar’):ti,ab,kw OR
			(‘lanifibranor’ OR ‘elafibranor’ OR ‘tetradecylthioacetic acid’):ti,ab,kw OR ((‘fatty acid metabolism” OR ‘fatty acid oxidation’) NEAR/5 (pharmacol*)):ti,ab,kw
	Standardised	EMB_PPAR_INDEXED	(‘peroxisome proliferator activated receptor’/exp OR ‘peroxisome proliferator activated receptor agonist’/exp OR ‘peroxisome proliferator activated receptor alpha agonist’/exp OR ‘peroxisome proliferator activated receptor delta agonist’/exp OR ‘peroxisome proliferator activated receptor gamma agonist’/exp OR “fibric acid derivative’/exp OR ‘glitazone derivative’/exp)
	Free-text or standardised	EMB_PPAR_COMBINED	EMB_PPAR_FREE OR EMB_PPAR_INDEXED
Kidney Disease (SC2) Adapted with modifications from Mihajlovic *et al* ^61^ and from Hickson *et al* ^62^	Free-text	EMB_KIDNEY_FREE	(‘kidney disease’ OR ‘kidney injury’ OR ‘kidney ischemia’ OR ‘kidney ischaemia’ OR ‘kidney diseases’ OR ‘kidney failure’ OR ‘kidney insufficiency’ OR ‘kidney fibrosis’ OR ‘renal disease’ OR ‘renal diseases’ OR ‘renal injury’ OR ‘renal ischemia’ OR ‘renal ischaemia’ OR ‘renal failure’ OR ‘renal insufficiency’ OR ‘renal fibrosis’ OR ‘acute kidney injury’ OR ‘acute renal injury’ OR ‘chronic kidney disease’ OR ‘chronic renal disease’ OR ‘diabetic kidney disease’ OR ‘diabetic renal disease’ OR ‘diabetic nephropathy’ OR ‘nephropathy’):ti,ab,kw
	Standardised	EMB_KIDNEY_INDEXED	‘kidney injury’/exp OR ‘artificial kidney’/exp OR ‘kidney disease’/exp OR ‘kidney failure’/exp OR ‘diabetic nephropathy’/exp
	Free-text or standardised	EMB_KIDNEY_COMBINED	EMB_KIDNEY_FREE OR EMB_KIDNEY_INDEXED
Animal Models (SC3)	Free-text	EMB_ANIMAL_FREE	SYRCLE EMBASE via Ovid animal search filter^64,65^ adapted for EMBASE via EMBASE.com
	Standardised	EMB_ANIMAL_INDEXED	SYRCLE EMBASE via Ovid animal search filter^64,65^ adapted for EMBASE via EMBASE.com
	Free-text or standardised	EMB_ANIMAL_COMBINED	EMB_ANIMAL_FREE OR EMB_ANIMAL_INDEXED
**Final Search (SC1 AND SC2 AND SC3)**	PPAR, kidney disease and animal model search terms	EMB_FINAL	EMB_PPAR_COMBINED AND EMB_KIDNEY_COMBINED AND EMB_ANIMAL_COMBINED AND (embase)/lim NOT ((embase)/lim AND (medline)/lim)

PPAR, peroxisome proliferator-activated receptor; SC, search component; SYRCLE, Systematic Review Centre for Laboratory Animal Experimentation.

**Table 4:** Web of Science Core Collection search strategy

Search component (number)	Search term type	Search alias	Search content
Pharmacological PPAR Targeting (SC1) Adapted with modifications from Liu and Wang^60^	Free-text	WOS_PPAR_FREE	TS = (((“peroxisome proliferator-activated receptor” OR ppar) NEAR/5 (agonist* OR modulator* OR stimulat* OR stimulant* OR activat*)) OR ((“peroxisome proliferator-activated receptor alpha” OR “ppar alpha” OR ppar-alpha OR pparalpha OR ppara OR nr1c1) NEAR/5 (agonist* OR modulator* OR stimulat* OR stimulant* OR activat*)) OR ((“peroxisome proliferator-activated receptor beta” OR “ppar beta” OR ppar-beta OR pparbeta OR pparb OR “peroxisome proliferator-activated receptor delta” OR “ppar delta” OR ppar-delta OR ppardelta OR ppard OR nr1c2) NEAR/5 (agonist* OR modulator* OR stimulat* OR stimulant* OR activat*)) OR
			((“peroxisome proliferator-activated receptor gamma” OR “ppar gamma” OR ppar-gamma OR ppargamma OR pparg OR nr1c3) NEAR/5 (agonist* OR modulator* OR stimulat* OR stimulant* OR activat*)) OR ((dual-PPAR OR “dual PPAR” OR PPAR-dual OR “PPAR dual” OR “dual-peroxisome proliferator-activated receptor” OR “dual peroxisome proliferator-activated receptor” OR “peroxisome proliferator-activated receptor-dual” OR “peroxisome proliferator-activated receptor dual”) NEAR/5 (agonist* OR modulator* OR stimulat* OR stimulant* OR activat*)) OR
			((pan-PPAR OR “pan PPAR” OR PPAR-pan OR “PPAR pan” OR “pan-peroxisome proliferator-activated receptor” OR “pan peroxisome proliferator-activated receptor” OR “peroxisome proliferator-activated receptor-pan” OR “peroxisome proliferator-activated receptor pan”) NEAR/5 (agonist* OR modulator* OR stimulat* OR stimulant* OR activat*)) OR
			(fibrate OR fibrates OR “fibric acid” OR “fibric acids” OR “fibric acid derivative” OR “fibric acid derivatives” OR bezafibrate OR “bezafibric acid” OR ciprofibrate OR “ciprofibric acid” OR clinofibrate OR “aluminium clofibrate” OR “aluminium clofibric acid” OR clofibrate OR “clofibric acid” OR clofibride OR “choline fenofibrate” OR “choline fenofibric acid” OR fenofibrate OR “fenofibric acid” OR gemfibrozil OR simfibrate) OR (WY-14,643 OR WY-14643 OR “WY 14,643” OR “WY 14643” OR WY14,643 OR WY14643 OR “pirinixic acid”) OR
			(“selective peroxisome proliferator-activated receptor modulat*” OR “selective PPAR modulat*” OR SPPAR OR SPPARM OR “SPPARM alpha” OR SPPARM-alpha OR SPPARMalpha OR SPPARMa OR pemafibrate OR K-877 OR K877) OR
			(thiazolidinedione OR thiazolidinediones OR glitazone OR glitazones OR pioglitazone OR rosiglitazone OR troglitazone OR netoglitazone OR rivoglitazone OR ciglitazone OR balaglitazone OR darglitazone OR edaglitazone OR englitazone OR lobeglitazone) OR
			(glitazar OR glitazars OR ragaglitazar OR tesaglitazar OR muraglitazar OR naveglitazar OR aleglitazar OR saroglitazar) OR
			(lanifibranor OR elafibranor OR “tetradecylthioacetic acid”) OR ((“fatty acid metabolism” OR “fatty acid oxidation”) NEAR/5 (pharmacol*)))
Kidney Disease (SC2) Adapted with modifications from Mihajlovic *et al* ^61^ and from Hickson *et al* ^62^	Free-text	WOS_KIDNEY_FREE	TS = (“kidney disease” OR “kidney injury” OR “kidney ischemia” OR “kidney ischaemia” OR “kidney diseases” OR “kidney failure” OR “kidney insufficiency” OR “kidney fibrosis” OR “renal disease” OR “renal diseases” OR “renal injury” OR “renal ischemia” OR “renal ischaemia” OR “renal failure” OR “renal insufficiency” OR “renal fibrosis” OR “acute kidney injury” OR “acute renal injury” OR “chronic kidney disease” OR “chronic renal disease” OR “diabetic kidney disease” OR “diabetic renal disease” OR “diabetic nephropathy” OR nephropathy)
Animal Models (SC3)	Free-text	WOS_ANIMAL_FREE	SYRCLE MEDLINE via PubMed animal search filter^63^ adapted for Web of Science Core Collection
Final Search (SC1 AND SC2 AND SC3)	PPAR, kidney disease, and animal model search terms	WOS_FINAL	WOS_PPAR_FREE AND WOS_KIDNEY_FREE AND WOS_ANIMAL_FREE

PPAR, peroxisome proliferator-activated receptor; SC, search component; SYRCLE, systematic review center for laboratory experimentation.

## Data Availability

All data relevant to the study are included in the article.

## Strengths and limitations of this study

► This systematic review protocol outlines a plan to collate, summarise and meta-analyse reports from preclinical studies evaluating the effect of targeted pharmacological activation of peroxisome proliferator-activated receptors (PPARs) on experimental renal injury.
► The efficacy of targeting individual PPAR isotypes as well as dual and pan-PPAR targeting will be evaluated through prespecified subgroup analyses.
► The findings will be applicable to a diverse range of human kidney diseases, including acute kidney injury and chronic kidney disease. The review will generate a robust record of the preclinical evidence base to help inform and guide future clinical studies evaluating pharmacological modulation of lipid metabolism as a therapeutic strategy in the management of renal disease.
► In order to promote transparent and accessible assessment of the study design and findings, the present protocol has been registered with Open Science Framework, https://osf.io/vcm4k/, and with PROSPERO.
► High levels of heterogeneity between studies may limit the generalisability of our findings, while reporting bias may result in overestimation of effect sizes in meta-analysis.
► Studies published in abstract format in conference proceedings for which no peer-reviewed full-text manuscript is available will be excluded, potentially limiting the inclusion of recently conducted studies in our review.
